# TRIM45 causes neuronal damage by aggravating microglia-mediated neuroinflammation upon cerebral ischemia and reperfusion injury

**DOI:** 10.1038/s12276-022-00734-y

**Published:** 2022-02-25

**Authors:** Qian Xia, Gaofeng Zhan, Meng Mao, Yin Zhao, Xing Li

**Affiliations:** 1 Department of Anesthesiology, Tongji Hospital, Tongji Medical College, Huazhong University of Science and Technology, Wuhan, 430030 Hubei Province China; 2 Department of Neurobiology, School of Basic Medicine, Tongji Medical College, Huazhong University of Science and Technology, Wuhan, 430030 Hubei Province China; 3 Department of Ophthalmology, Tongji Hospital, Tongji Medical College, Huazhong University of Science and Technology, Wuhan, 430030 Hubei Province China

**Keywords:** Cell death in the nervous system, Stroke

## Abstract

Excessive and unresolved neuroinflammation is a key component of the pathological cascade in brain injuries such as ischemic stroke. Tripartite motif-containing 45 (TRIM45) is a ubiquitin E3 ligase involved in various critical biological processes. However, the role of TRIM45 in cerebral ischemia remains unknown. Here, we found that the TRIM45 protein was highly expressed in the peri-infarct areas of mice subjected to cerebral ischemia and reperfusion injury induced by middle cerebral artery occlusion. This study systemically evaluated the putative role of TRIM45 in the regulation of neuroinflammation during ischemic injury and the potential underlying mechanisms. We found that TRIM45 knockdown significantly decreased proinflammatory cytokine and chemokine production in primary cultured microglia challenged with oxygen-glucose deprivation and reoxygenation (OGD/R) treatment. Mechanistically, we demonstrated that TRIM45 constitutively interacted with TAB2 and consequently facilitated the Lys-63-linked polyubiquitination of TAB2, leading to the formation of the TAB1–TAK1–TAB2 complex and activation of TAK1, which was ultimately followed by activation of the nuclear factor-kappa B (NF-κB) signaling pathway. In an in vitro coculture Transwell system, downregulation of TRIM45 expression also inhibited the OGD/R-induced activation of microglia and alleviated neuronal apoptosis. More importantly, microglia-specific knockdown of TRIM45 in mice significantly reduced the infarct size, mitigated neurological deficit scores, and improved cognitive function after ischemic stroke. Taken together, our study reveals that the TRIM45–TAB2 axis is a crucial checkpoint that controls NF-κB signaling in microglia during cerebral ischemia and reperfusion injury. Therefore, targeting TRIM45 may be an attractive therapeutic strategy.

## Stroke: Possible therapeutic target for subsequent brain inflammation

Therapies targeting a protein found at high levels following stroke could help reduce subsequent brain inflammation and damage to neurons. The release of cytokines and chemokines, immune system signaling molecules, in the brain following a stroke exacerbates inflammation and can cause extensive damage, even after restoration of blood flow. Xing Li and co-workers at Huazhong University of Science and Technology, Wuhan, China, examined the role of the TRIM45 protein following stroke in mice. The brain’s immune system cells, microglia, activate inflammatory proteins to protect the brain from injury. The team found that stroke triggers the increase of TRIM45 expression in microglia. TRIM45 interacts with another protein to stimulate signalling cascades that result in inflammation and neuronal damage. Blocking TRIM45 expression significantly decreased cytokine production in the mice, suggesting the protein may be a valuable therapeutic target.

## Introduction

Cerebral ischemia is a widespread leading cause of death and disability, and it is mainly caused by decreased blood flow to the brain and consequently results in brain tissue damage^1^. Previous reports have shown that neuroprotective strategies targeting neurons alone cannot produce better outcomes after ischemia-reperfusion (I/R) injury^2^. Moreover, cumulative studies have concluded that the cytokine-dependent microenvironment plays a vital role in the progression of ischemic stroke^3^. Harmful molecules are released after the ischemic period, resulting in sterile inflammation of the brain with the release of cytokines and chemokines^4^. New treatment strategies to promote tissue and functional repairs and thus regulate the microenvironment have recently attracted increased attention.

Microglia represent a likely primary source of inflammatory mediators in the central nervous system^5^. Sudden blockage of cerebral blood flow leads to energy exhaustion and neuronal injury, which triggers an immune response, ultimately leading to the activation of inflammatory cells^6,7^. First, gene expression in damaged neurons is rapidly changed, and multiple factors, such as ATP and glutamic acid, are produced, which stimulate the activation and migration of nearby microglia to protect the brain from ischemic stroke^8^. In addition, excessively activated microglia activate different inflammatory pathways, such as the nuclear factor-κB (NF-κB) pathway, that trigger the release of a large variety of proinflammatory mediators, including IL-1β, IL-6, and TNF-α, which can lead to acute inflammatory reactions. The excessive production of inflammatory cytokines can exacerbate damage to neighboring neurons and result in delayed deterioration of ischemic tissue^9,10^. Accordingly, the identification of pivotal regulators that target these signaling molecules and attenuate the production of inflammatory factors by microglia would prevent neuronal death after ischemic stroke.

Previous research has shown that the tripartite motif-containing (TRIM) protein family, characterized by a RING finger, B-box zinc finger, and coiled-coil domain with conventional ubiquitin E3 ligase activity, plays an indispensable role in regulating many biological processes, such as the inflammatory response, innate immunity, cell proliferation and apoptosis^11,12^. Studies from different research groups have revealed the functions of TRIM8/9/47/72 in ischemic stroke^13–16^. As a member of the TRIM family, TRIM45 contains a filamin-type immunoglobulin (FLMN) domain in its carboxy-terminal region^17^. TRIM45 was reported to be a tumor suppressor in the brain via its E3 ligase activity by stabilizing p53 through K63-linked ubiquitination^18^. Moreover, TRIM45 regulates cell proliferation by inhibiting the NF-κB signaling pathway^19^. TRIM45 is highly expressed in the brains of human adult and embryonic tissues^20^. However, whether and how TRIM45 plays a role in cerebral ischemia remains to be investigated.

In this study, we found that ischemic stroke significantly upregulated TRIM45 expression in microglia. We identified microglial TRIM45 as a key modulator of ischemic signaling cascades that promoted the development of cerebral I/R-induced inflammatory responses and neuronal damage both in vivo and in vitro. Mechanistically, TRIM45 directly interacts with TAB2 by promoting K63-linked polyubiquitination of TAB2, thereby inducing TAB1–TAK1–TAB2 complex formation and promoting TAK1 autophosphorylation. Activated TAK1 then stimulates NF-κB-mediated inflammation and neuronal cell death after cerebral I/R injury. Together, these findings suggest that TRIM45 functions as a novel signaling regulator in ischemic stroke injury and may be a promising approach to protect neuronal function against ischemic stroke-related injury.

## Materials and methods

### Animals

C57BL/6 J male mice (weighing 22–25 g, aged 8 weeks) were obtained from Beijing Vital River Laboratory Animal Technology Co., Ltd. (Beijing, China). Cx3cr-Cre mice (C57BL/6J background) were purchased from Jackson Laboratory (JAX stock No: 025524). Mice were housed in a special breeding box that was placed in a quiet room. The breeding environment was kept under a 12–12 h light-dark cycle, controlled temperature (approximately 22 °C), and 40–60% relative humidity with water and food ad libitum. All animal experiments were approved by the Institutional Animal Care and Use Committee of Huazhong University of Science and Technology. The study was conducted according to the IMPROVE guidelines^21^. The protocols and details of this report were in accordance with the ARRIVE guidelines^22^. The number of animals for each group was predetermined according to numbers reported in published studies or our prior experiment, and accurate animal numbers are given in the figure legends. All animals were randomized for the research and procedures. The operator was blinded to the experimental procedures and data analysis throughout the experiment.

### Transient focal cerebral ischemia

Transient focal cerebral ischemia was produced by transient occlusion of the left middle cerebral artery (MCAO) using the intraluminal filament technique as previously described^23^. In general, a feedback control heating system was used to maintain the rectal temperature at 37 °C. Male mice aged 8 weeks (22–25 g) were anesthetized with 350 mg/kg 4% chloral hydrate (i.p.). A midline neck incision was performed to expose the left external carotid artery (ECA), internal carotid artery (ICA) and common carotid artery (CCA). Next, the distal end of the CCA was ligated. The ECA was ligated with two surgical nylon monofilaments: one was at the distal end of the ECA, and the other was the bifurcation of the ICA and the ECA. Then, we made a small incision at the tube between the ECA ligatures. A 2-cm long nylon filament (diameter 0.25 ± 0.03 mm) was gently inserted into the lumen of the ICA from the ECA to the approximately marked position with a red mark at 1 cm. Cerebral blood flow was monitored by laser Doppler flowmetry (PeriFlux System 5000; Perimed, Sweden). I/R was defined as a minimum of 75% decrease in cerebral blood flow at the onset of ischemia and a return to 50% of baseline blood flow measurement. Mice not achieving these criteria were excluded from the study. After ischemia for 1 h, the clamp was withdrawn for reperfusion. For the sham group, mice were subjected to the same surgical procedure without vaso-occlusion.

### Cell culture, transfection, oxygen-glucose deprivation and reoxygenation (OGD/R) procedure

Primary cultured cortical neurons were prepared from embryonic Day 17 mice as described previously^24^. The separated cells were placed in 6-well culture plates at a density of 1 × 10^7^ cells per well. Primary mouse microglial cells were prepared from 1- to 2-day-old neonatal C57BL/6 J mouse brains. Briefly, cerebral cortices were gently separated, and a 70-µm pore filter was used to filter the cell suspension. Finally, the cells were incubated in vented T75 flasks (Corning, Shanghai, China) at 37 °C and resuspended in high-glucose Dulbecco’s modified Eagle’s medium (DMEM, Thermo Fisher Scientific, Waltham, MA, USA) containing 10% fetal bovine serum (FBS, Gibco, Gaithersburg, MD, USA). Primary microglial cells were collected from the culture after dissection for 10–14 days by shaking at 400 rpm for 6 h on a 37 °C rotary oscillator. Then, neurons and microglia were used for the following in vitro experiments. A highly enriched population of microglia was isolated from adult mice by Percoll density centrifugation using a protocol as we described previously^25^.

HEK293T cells were cultured in high-glucose DMEM supplemented with 10% FBS and incubated at 37 °C with 5% CO_2_. Lipofectamine 3000 (Invitrogen, Carlsbad, CA, USA) was utilized to transfect the indicated cells according to the manufacturer’s instructions. We established an in vitro model of ischemic stroke by exposing cultured cells to oxygen-glucose deprivation and reoxygenation (OGD/R) as previously described^23^. Briefly, the cultivation media was substituted with glucose-free DMEM and incubated in 5% CO_2_ and 95% N_2_ at 37 °C. After 60 min, the medium was replaced with high-glucose DMEM, and the cells were returned to a normoxic conditional incubator with 5% CO_2_ and reoxygenated. After reoxygenation for 24 h, the cells were collected for the following studies.

### Lentiviral production and transfection in cells

shRNA synthesis was based on a previous study^26^. Briefly, the target sequence for mouse *TRIM45* (GenBank NM_001165952.1) was cloned into the pGC-LV vector. pGC-LV-TRIM45 shRNA, pHelper 1.0, and pHelper 2.0 were cotransfected into HEK293T cells according to standard protocols. LV-shTRIM45 was harvested 72 h later, and LV-scramble was used as the reference control virus. Viral particles were resuspended in serum-free medium and stored at −80 °C. The interference sequence was as follows: no. 1 was 5′-GCTCAGGAAGCTGAATAAAGT-3′, no. 2 was 5′-GGTCTTGCTGTGGGAAGTTCA-3′, and no. 3 was 5′-GGTGGAGTGAAGGCTTTAACG-3′.

### AAV viral vectors transduction in mice

AAV2/6 viruses encoding U6-DIO-Scramble-EGFP and U6-DIO-TRIM45 shRNA-EGFP were injected into Cx3cr-Cre mice to induce microglia-specific TRIM45 knockdown. The effective interference sequence of mouse *TRIM45* (GenBank NM_001165952.1) shRNA in AAV-TRIM45 shRNA was 5′-GGTGGAGTGAAGGCTTTAACG-3′. Scramble sequences were used to construct a nontargeting control virus. These virus titers were 2–3 × 10^12^ vg/ml. We divided the viral vector into aliquots and stored it at −80 °C until use. Stereotactic surgery was performed to deliver the AAV vector. Briefly, male mice aged 11–12 weeks (25–30 g) were anesthetized with 350 mg/kg 4% chloral hydrate (i.p.) and fixed on a stereotaxic instrument (RWD Life Science, Shenzhen, China). A burr hole was used to perforate the skull, and a stepper motor-driven microinjector (Hamilton, Reno, NV, USA) was used to inject 500 nL of virus solution at each injection site into the hippocampal CA1 area, cerebral cortex, and striatum of the left hemisphere. The rate was 50 nL/min. The stereotaxic coordinates were as follows: hippocampal CA1 region (AP: −2.00 mm, ML: −1.55 mm, DV: −1.55 mm), cerebral cortex region (AP: 0.00 mm, ML: −2.05 mm, DV: −1.50 mm), and striatum region (AP: 0.00 mm, ML: −2.05 mm, DV: −3.50 mm).

### Immunoblot analysis

Total proteins were harvested from cells with ice-cold RIPA buffer (Beyotime Biotechnology, Shanghai, China). Lysates were centrifuged at 12,000 × *g* at 4 °C for 15 min, and then, the protein samples were heated at 98 °C for 8 min. SDS-PAGE gels (10% or 12%) were utilized to separate the proteins, and then, the proteins were transferred onto a polyvinylidene fluoride membrane (PVDF; Roche). After 1 h of incubation with 5% fat-free milk in TBST, the membranes were probed with the primary antibodies of interest overnight at 4 °C, followed by HRP-conjugated secondary antibodies for 1 h at room temperature. Finally, protein bands were developed by chemiluminescence detection (ECL; Thermo Fisher Scientific), and then, the band densitometry was analyzed with ImageJ software. All Western blot data are expressed as the ratio of the levels of the protein of interest and α-tubulin or β-actin. All antibodies are listed in Supplementary Table 1.

### RNA extraction, reverse transcription, and quantitative real-time PCR

RNA was collected from cell samples with TRIzol reagent (Invitrogen) according to the product manual and then quantified with a spectrophotometer. Next, complementary DNA was synthesized from 1 μg of total RNA via the ReverTra Ace-α-TM First Strand cDNA Synthesis Kit (Toyobo, Osaka, Japan). The Quanti-Fast™ SYBR® Green PCR Kit (Applied Biosystems, Foster City, CA, USA) was used for quantitative real-time PCR (qRT-PCR) analysis according to the manufacturer’s instructions. The 2^−ΔΔCt^ method was utilized to analyze gene expression, and relative mRNA expression values were normalized to β-actin mRNA levels. Primer sequences are listed in Supplementary Table 2.

### 2, 3, 5-triphenyltetrazolium chloride (TTC) staining

2, 3, 5-triphenyltetrazolium chloride (TTC) staining was conducted as we described previously^27^. Mice were euthanized after MCAO surgery, and TTC staining was conducted to examine the ischemic infarct. The brain was rapidly extracted and frozen for 10 min at −20 °C. The brains of mice were cut into six 2-mm thick slices from the frontal tips with a mouse brain matrix (RWD Life Science). Subsequently, sections were stained with 1% TTC (Sigma-Aldrich) at 37 °C for 30 min. Next, sections were submerged and fixed in 4% PFA. The ischemic infarct area of each section was analyzed using ImageJ software (NIH, Baltimore, MD, USA). The ischemic infarct size was summed and is shown as follows: Infarct size (%) = (contralateral area-ipsilateral noninfarct area)/contralateral area × 100%.

### Morris water maze (MWM) test

The MWM test was conducted to detect spatial learning and memory as described previously^28^. Briefly, the water maze contained a 120-cm diameter circular water tank filled with 60 cm high opaque water at a temperature of 22 ± 2 °C and a 6 cm diameter round platform, and the platform was immersed 1 cm below the water surface. Different shapes were posted along the tank wall to serve as spatial reference tips. Above the maze, a digital tracking device (Xinruan Information Technology, Shanghai, China) was installed to record the swimming trajectory in the water maze. Mice were tested at the same time each day. During the latency trials, underwater platform training was conducted for 6 consecutive days, and each stage included 4 trials. In each test, animals were released from the tank wall and permitted to search and stand on the hidden platform during the 60 s test period. If the animals did not find the platform within the specified time, it was guided to the platform and remained on the platform for 15 s to facilitate learning and directional memory. Probe testing was performed 24 h after the training. In the probe test, the platform was removed, and the mouse’s performance was recorded for 60 s. The time spent in the target quadrant, the latency to reach the platform, the distance to reach the platform, and the numbers across the platform were recorded automatically for subsequent analysis.

### Novel object recognition

The novel object recognition test was conducted as described previously with minor modifications^25^. Before the test, mice were placed in the test room and acclimated for 30 min for 2 consecutive days. On the first day of the experiment, each mouse was placed in an open field apparatus (50 × 50 × 50 cm) with two identical objects (familiar object), which were placed on one side of the open field box and permitted to freely explore the objects for 5 min. Subsequently, after 1 h, a novel object replaced one of the familiar objects, and the animals were allowed to explore for 5 min. A digital video-tracking system (Xinruan Information Technology) was used to record the time spent exploring the familiar and novel objects. Between each experiment, 75% ethanol was used to clean the chamber and objects to remove olfactory stimuli. The results are expressed as contacting the object (tail only excluded) or facing the object (distance <2 cm). For analysis of CA1-dependent cognition, the discrimination ratio of time spent with the novel object to the total exploration time was analyzed, and a higher preference for new objects was rated as complete spatial recognition memory.

### Statistical analysis

GraphPad Prism 8.0 (GraphPad Software, Inc., San Diego, CA, USA) was used to perform all statistical tests. All the results are shown as the mean ± S.E.M. from at least three independent experiments. The experiments with only 2 groups were analyzed with the unpaired two-tailed Student’s *t* test. Single-factor experiments with >2 groups were analyzed with one-way analysis of variance (ANOVA) with Dunnett’s post-hoc test. The double factor experiments were analyzed with two-way ANOVA followed by multiple comparisons with Tukey’s post-hoc test. In the water maze test, escape latency during the training phase (6 days) was analyzed with repeated-measures (RM) ANOVA. *p* < 0.05 was considered statistically significant, and n.s. represents no significant.

## Results

### TRIM45 expression in microglia is upregulated after ischemic stroke

**Fig. 1:**
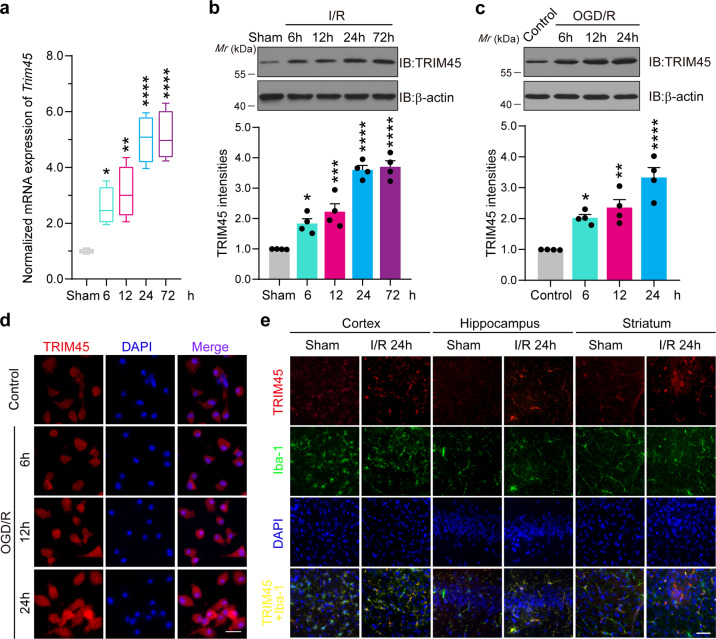
TRIM45 expression was upregulated after cerebral I/R injury. **a** qRT-PCR assay indicating TRIM45 expression in cerebral tissues at the indicated time points post-middle cerebral artery occlusion/reperfusion; *n* = 4 per time point. **b** Mouse brain homogenates were extracted at the indicated time points following cerebral I/R injury. Western blot assay (above) and quantification (blow) showing TRIM45 expression; *n* = 4 per time point. **c** Homogenates of primary microglia subjected to oxygen and glucose deprivation (OGD) and reoxygenation at the indicated time points were extracted. Western blot assay (above) and quantification (blow) showing TRIM45 expression; *n* = 4 per time point. **d** Primary microglia were subjected to oxygen and glucose deprivation (OGD) and reoxygenation at the indicated time points. Immunofluorescence indicating TRIM45 expression; TRIM45 (red), and 4’,6-diamidino-2-phenylindole (DAPI; blue, nuclei). Scale bars, 20 μm. **e** Immunofluorescence indicating TRIM45 expression in the cerebral cortex, hippocampal CA1 region, and striatum of adult mice subjected to MCAO surgery for 1 h and reperfusion for 24 h; TRIM45 (red), Iba-1 (green) and nuclei (DAPI), scale bars, 40 μm. Data are presented as the mean ± S.E.M. and were analyzed by one-way ANOVA followed by Dunnett’s post hoc test. *P < 0.05, **P < 0.01, ***P < 0.001 and ****P < 0.0001.

To evaluate the role of TRIM45 in cerebral I/R injury, we examined TRIM45 expression in mouse brain tissues. Therefore, an experimental stroke model was established by MCAO surgery, and qRT-PCR indicated that the *TRIM45* mRNA levels increased at 6 h and reached a peak at 24 h after ischemic stroke (Fig. 1a). Moreover, immunoblot analysis showed the same trend (Fig. 1b). Next, to examine which cell type exhibited upregulated TRIM45 expression after MCAO, we isolated and cultured microglia, astrocytes, and neurons from the brains of neonatal mice and subjected these cells to OGD/R. qRT-PCR analysis showed that only microglia exhibited elevated TRIM45 expression (Supplementary Fig. 1). Consistent with these observations, immunoblotting revealed that TRIM45 protein levels in primary microglia progressively increased with longer periods of OGD/R (Fig. 1c). These results were also confirmed by immunofluorescence analysis (Fig. 1d). Moreover, the immunofluorescence results revealed that TRIM45 was increased after stroke in vivo (Fig. 1e). Collectively, these data suggest that TRIM45 expression is significantly upregulated after ischemic stroke and that activated microglia are the main source of upregulated TRIM45 expression.

### TRIM45 promotes NF-κB proinflammatory signaling activation in microglial cells after OGD/R

**Fig. 2:**
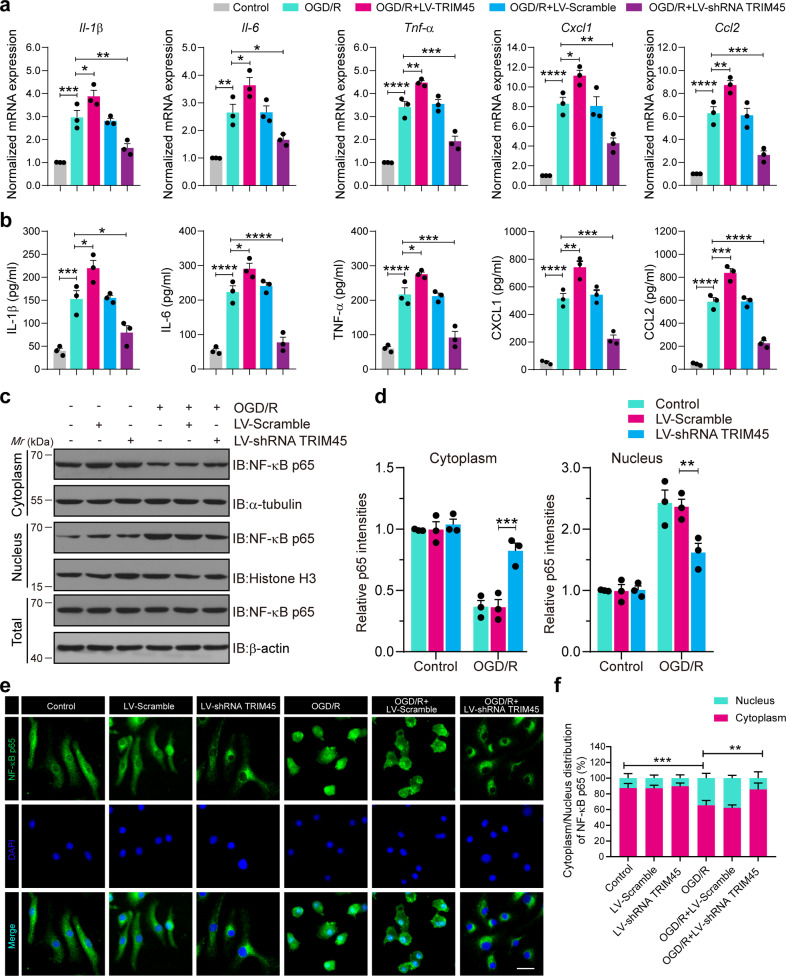
TRIM45 enhances the NF-κB signaling pathway through TAB2 and TAK1. **a** qRT-PCR showing the mRNA levels of proinflammatory genes and chemokines, such as *IL-1β, IL-6, TNF-α, Cxcl1*, and *Ccl2*, in primary cultured microglia transfected with lentivirus encoding shTRIM45 or the scramble control and then subjected to OGD/R after 48 h. **b** The secretion levels of IL-1β, IL-6, TNF-α, Cxcl1, and Ccl2 were determined by ELISAs of primary cultured microglia. **c**, **d** Western blot assay (**c**) and quantification (**d**) indicating NF-κB p65 levels in the cytoplasm and nucleus of primary cultured microglia. **e**, **f** Primary cultured microglial cells were transfected with lentivirus encoding shTRIM45 or the scramble control and then subjected to OGD/R after 48 h. Immunofluorescence assay (**e**) and quantification (**f**) indicated NF-κB p65 levels in the cytoplasm and nucleus. NF-κB p65 (green), nuclei (DAPI), Scale bars, 40 μm. Data in the (**d**, **f**) were analyzed by two-way ANOVA followed by Tukey’s post hoc test, and all others were analyzed by one-way ANOVA followed by Dunnett’s post hoc test. Data are presented as the mean ± S.E.M. from at least three independent experiments. n.s. for *P* > 0.05, *P < 0.05, **P < 0.01, ***P < 0.001 and ****P < 0.0001.

Inflammation plays a critical role in I/R injury^29^. Given the increased expression of TRIM45 in microglia following stroke, we investigated the functional role of TRIM45 in the microglia-mediated inflammatory response. First, immunoblotting results revealed that TRIM45 shRNA#3 clearly exhibited the best interference efficiency (Supplementary Fig. 2a, b), and TRIM45 shRNA#3 was used for the following experiment. Then, lentiviruses encoding TRIM45 (LV-TRIM45), LV-shRNA TRIM45 or scramble were used to infect primary cultured microglia. We conducted qRT-PCR to evaluate the mRNA levels of proinflammatory cytokines and chemokines, such as *IL-1β, IL-6, TNF-α, Cxcl1*, and *Ccl2*. The results demonstrated that TRIM45 overexpression significantly upregulated the levels of these proinflammatory cytokines and chemokines, while downregulation of TRIM45 expression showed the opposite effect (Fig. 2a). In addition, the levels of the indicated proinflammatory cytokines and chemokines secreted by primary cultured microglia were higher in the TRIM45 overexpression group but lower in the TRIM45 knockdown group than in the control group under OGD/R conditions, as determined by ELISAs (Fig. 2b).

To investigate the underlying molecular mechanism, we examined the activation of NF-κB signaling pathways, which play critical roles in the microglia-mediated inflammatory response^30^. Accordingly, increasing NF-κB p65 nuclear translocation is the hallmark of NF-κB activation. Immunoblotting analysis showed that TRIM45 knockdown clearly reduced the levels of NF-κB p65 in the nucleus under OGD/R conditions (Fig. 2c, d). Furthermore, these results were further confirmed by immunofluorescence analysis (Fig. 2e, f). Next, we used the dual luciferase reporter assay to measure p65 transcriptional activity. The results suggested that TRIM45 knockdown decreased p65 transcriptional activity (Supplementary Fig. 3a), while TRIM45 overexpression increased p65 transcriptional activation, as expected (Supplementary Fig. 3b). To further verify that TRIM45 mediates the inflammatory response associated with the NF-κB signaling pathway, we pretreated microglial cells with 100 μM PDTC, an inhibitor of the NF-κB signaling pathway, for 1 h after infection with LV-TRIM45 for 48 h and then subjected them to OGD/R. The cell supernatants were collected and assayed by ELISAs. The results indicated that TRIM45 overexpression increased the secretion of IL-1, IL-6, and TNF-α in microglial cells after OGD/R. However, pretreatment with PDTC inhibited this effect (Supplementary Fig. 3c–e). Altogether, these results suggest that TRIM45 increases proinflammatory cytokine and chemokine levels via the NF-κB signaling pathway in microglial cells after OGD/R.

### TRIM45 directly interacts with TAB2 through its RING domain after OGD/R

**Fig. 3:**
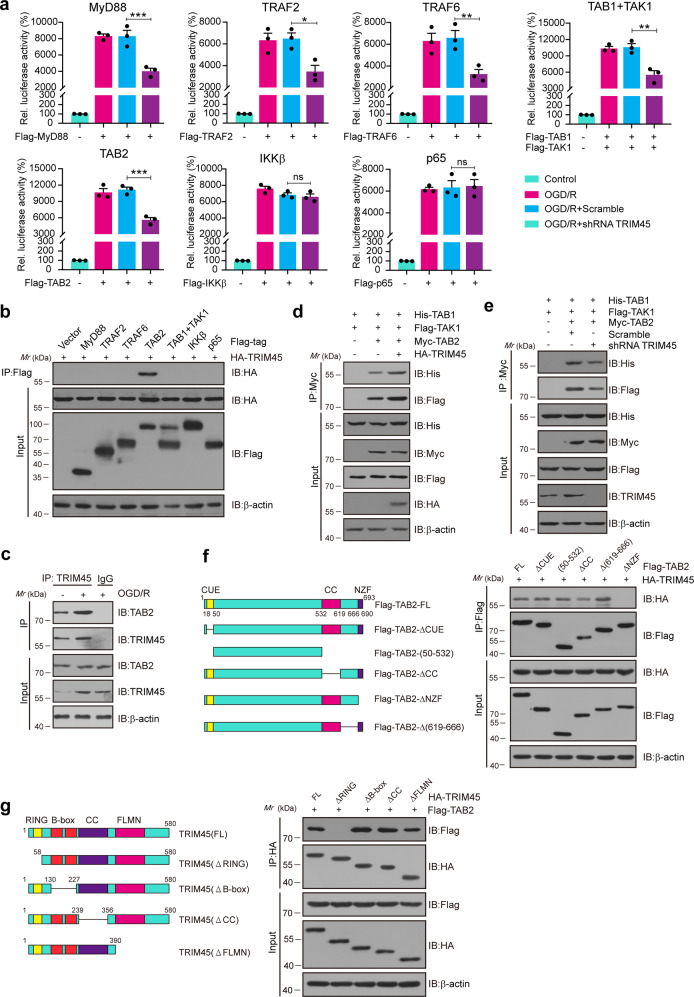
TRIM45 directly interacts with TAB2 after OGD/R. **a** HEK293T cells were transfected with plasmids encoding empty vector, MyD88, TRAF2, TRAF6, TAB1/TAK1, TAB2, IKKβ, or p65 together with the luciferase reporter for NF-κB along with TRIM45 shRNA. The NF-κB luciferase activity was analyzed. **b** HEK293T cells were transfected with plasmids encoding empty vector, Flag-tagged MyD88, TRAF2, TRAF6, TAB1/TAK1, TAB2, IKKβ, or p65 together with HA-TRIM45. Coimmunoprecipitation (Co-IP) and IB analysis showing the various combinations (above lanes). **c** Primary cultured microglia were subjected to OGD/R. Co-IP analysis indicating the combinations of TRIM45 and TAB2. **d** Co-IP analysis showing the interaction between TAB2 and TAK1, or TAB1 in HEK293T cells subjected to TRIM45 overexpression after OGD/R. **e** Co-IP analysis showing the interaction between TAB2 and TAK1, or TAB1 in HEK293T cells subjected to TRIM45 knockdown after OGD/R. **f** Co-IP analysis showing the various combinations (right) in HEK293T cells transfected with TAB2 deletion mutants (left) along with HA-TRIM45. CUE, ubiquitin conjugated enzyme binding domain; CC, a coiled-coil domain; NZF, zinc finger domain. Data are representative of three independent experiments. **g** Co-IP analysis showing the various combinations (right) in HEK293T cells transfected with TIRM45 deletion mutants (left) along with Flag-TAB2. B-box, B-box domain; RING, a RING-finger domain; CC, a coiled-coil domain; FLMN, a filamin-type immunoglobulin domain. Data are presented as the mean ± S.E.M. and were analyzed by one-way ANOVA followed by Dunnett’s post hoc test. n.s. for *P* > 0.05, *P < 0.05, **P < 0.01, ***P < 0.001.

We then explored the precise mechanisms by which TRIM45 regulated OGD/R-mediated NF-κB activation. It is widely accepted that NF-κB activation is involved in several major steps^31^. In response to proinflammatory stimuli, adaptors such as MyD88, TRAF2, TRAF6, TAB1/TAK1, or TAB2 induce IKK complex activation, followed by IκBα phosphorylation and subsequent proteasomal degradation, ultimately resulting in p65 nuclear translocation, which then activates inflammatory gene transcription^32,33^. The luciferase reporter assay results suggested that TRIM45 ablation decreased the NF-κB reporter activity induced by MyD88, TRAF2, TRAF6, TAB1/TAK1, and TAB2 but not IKKβ or p65 (Fig. 3a), indicating that downregulated TRIM45 expression suppresses the NF-κB signaling pathway upstream of IKK, most likely through targeting of the TAK1–TAB1–TAB2 complex. Next, we investigated whether TRIM45 directly binds to these signaling proteins within the NF-κB pathway. Interestingly, coimmunoprecipitation (Co-IP) assays confirmed that TRIM45 markedly interacted with TAB2 but not MyD88, TRAF2, TRAF6, TAB1/TAK1, IKKβ, or p65 (Fig. 3b). Furthermore, co-IP assays indicated that the interaction between TRIM45 and TAB2 markedly increased in microglial cells after OGD/R (Fig. 3c). Altogether, these data revealed that TRIM45 activates the NF-κB signaling pathway, probably through TAB2.

Previous studies have found that the formation of the TAK1–TAB1–TAB2 complex is important for NF-κB activation. It has been reported that TAB2 and TAB1 function as adaptor proteins that bind to TAK1, thereby inducing TAK1 activation and promoting the subsequent activation of IKK^34^. Therefore, we aimed to further explore the effect of TRIM45 on the association of TAB2, TAK1, and TAB1 and found that TRIM45 overexpression markedly enhanced the interaction between TAB2 and TAK1 or TAB1 in vitro (Fig. 3d). Next, we examined the interference efficiency of TRIM45 shRNAs in HEK293T cells, and the immunoblot results showed that TRIM45 shRNA#2 clearly decreased the TRIM45 protein level (Supplementary Fig. 4). An effective interference plasmid was used in the following experiments. As expected, TRIM45 knockdown markedly decreased the interaction between TAB2 and TAK1 or TAB1 (Fig. 3e). These results suggested that OGD/R induced the interaction between TRIM45 and TAB2 and indirectly promoted the binding of TAK1 and TAB1 to form the TAK1–TAB1–TAB2 complex.

To distinguish the domain of TAB2 required for its interaction with TRIM45, we generated five TAB2 truncation mutants (Fig. 3f, left panel). Some deletion mutants of TAB2 together with HA-TRIM45 were cotransfected into HEK293T cells. We found that the novel zinc finger domain (NZF) was essential for its binding to TRIM45 (Fig. 3f, right panel). Next, we elucidated the domain of TRIM45 responsible for its interaction with TAB2. We then generated four TRIM45 deletion mutants, and co-IP assays confirmed that the RING domain was critical for the interaction with TAB2 (Fig. 3g). Altogether, these data suggested that TRIM45 directly interacts with TAB2 through its RING domain.

### TRIM45 enhances TAK1 phosphorylation levels in a TAB2-dependent manner

**Fig. 4:**
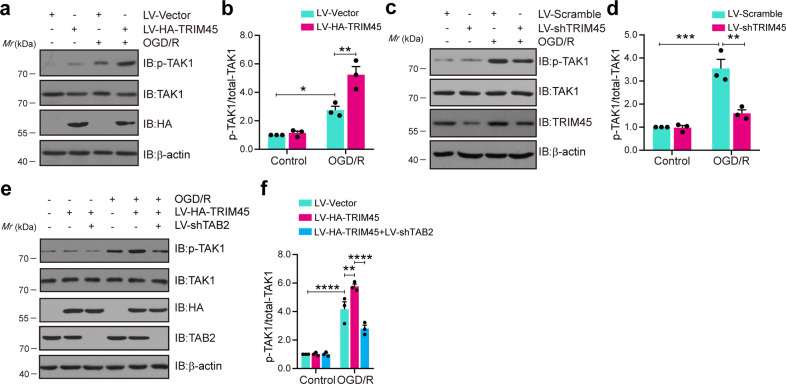
TRIM45 enhances TAK1 phosphorylation levels in a TAB2-dependent manner. **a**, **b** Western blot assays (**a**) and quantification (**b**) showing p-TAK1 levels in primary microglial cells subjected to LV-TRIM45 infection after OGD/R. **c**, **d** Western blot assays (**c**) and quantification (**d**) showing p-TAK1 levels in primary microglial cells subjected to LV-shTRIM45 infection after OGD/R. **e**, **f** Primary microglial cells were infected with LV-TRIM45 together with LV-shTAB2. Western blot assays (**e**) and quantification (**f**) showing p-TAK1 levels. Data are presented as the mean ± S.E.M. from at least three independent experiments and were analyzed by two-way ANOVA followed by Tukey’s post hoc test. ns, no significance. *P < 0.05, **P < 0.01, ***P < 0.001, ****P < 0.0001.

Some research has reported that the interaction of TAK1 and TAB2 facilitates autophosphorylation of TAK1 (Thr178, Thr184, Thr187, and Ser192) within the kinase activation loop, which leads to NF-κB pathway activation^34–37^. Therefore, we aimed to determine the effect of TRIM45 on TAK1 phosphorylation. First, immunoblotting assays showed that upregulated TRIM45 expression increased the phosphorylation of TAK1 (p-TAK1) in primary microglial cells subjected to OGD/R (Fig. 4a, b). Additionally, TRIM45 knockdown severely impaired the p-TAK1 level after OGD/R (Fig. 4c, d). Therefore, we sought to examine whether the effect of TRIM45 on TAK1 phosphorylation was related to TAB2. Immunoblotting assays revealed that TRIM45 overexpression increased TAK1 phosphorylation, but this effect was reversed by TAB2 knockdown (Fig. 4e, f). Altogether, these findings demonstrated that TRIM45 regulates TAK1 phosphorylation in a TAB2-dependent manner.

### TRIM45 promotes the association of TAK1 and TAB2 based on its E3 ligase activity

**Fig. 5:**
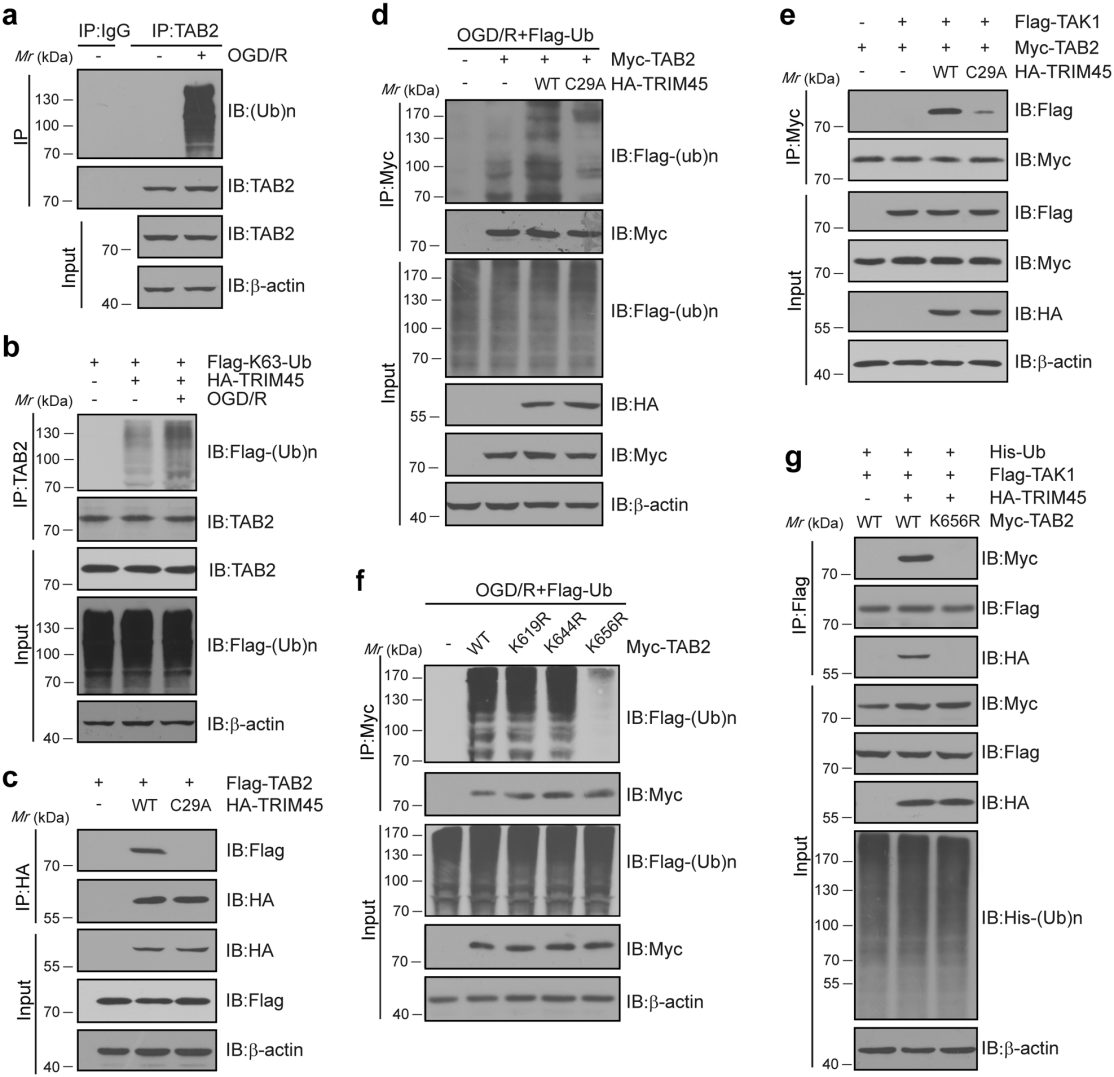
TRIM45 regulates NF-κB signaling dependent on its E3 ligase activity. **a** Co-IP analysis indicating TAK2 ubiquitination in primary microglial cells subjected to OGD/R. The cell extracts were collected for IP with anti-TAB2 beads, followed by IB analysis with the indicated antibodies. **b** HEK293T cells were transfected with plasmids encoding HA-TRIM45 and Flag-tagged K63-only ubiquitin (Flag-K63-Ub) with or without OGD/R treatment. The cell extracts were collected for IP with anti-TAB2 beads, followed by IB analysis with the indicated antibodies. **c** HEK293T cells were transfected with plasmids encoding Flag-TAB2 and HA-tagged wild-type TRIM45 or TRIM45-C29A. The cell extracts were collected for IP with anti-HA beads, followed by IB analysis with the indicated antibodies. **d** HEK293T cells were transfected with plasmids encoding Myc-TAB2, Flag-Ub and HA-tagged wild-type TRIM45 or TRIM45-C29A and subjected to OGD/R. The cell extracts were collected for IP with anti-Myc beads, followed by IB analysis with the indicated antibodies. **e** HEK293T cells were transfected with plasmids encoding Myc-TAB2, Flag-TAK1 and HA-tagged wild-type TRIM45 or TRIM45-C29A. The cell extracts were collected for IP with anti-Myc beads, followed by IB analysis with the indicated antibodies. **f** HEK293T cells were transfected with plasmids encoding Myc-TAB2-K619R, K644R or K656R together with Flag-Ub and subjected to OGD/R. The cell extracts were collected for IP with anti-Myc beads, followed by IB analysis with the indicated antibodies. **g** HEK293T cells were transfected with plasmids encoding His-Ub, Flag-TAK1, and HA-TRIM45 together with Myc-TAB2 or K656R. The cell extracts were collected for IP with anti-Flag beads, followed by IB analysis with the indicated antibodies. Data are representative of at least three independent experiments.

As the RING domain of TRIM45 is crucial for its function^38^, we explored whether TRIM45 exerts its function via its E3 ligase activity. First, we found that OGD/R induced TAB2 polyubiquitination in microglia (Fig. 5a). In addition, HEK293T cells were transfected with HA-tagged TRIM45 together with Flag-tagged wild-type ubiquitin (Supplementary Fig. 5a), K63-only ubiquitin (Fig. 5b) or K48-only ubiquitin plasmids (Supplementary Fig. 5b). The results indicated that TRIM45 induced TAB2 polyubiquitination, but only with a K63 linkage and not with a K48 linkage. Consistent with the research that has been reported, polyubiquitin chain formation through K48 of ubiquitin promoted modified protein degradation; however, K63-linked polyubiquitin chains normally do not lead to substrate degradation but mediate low-affinity binding of other proteins that contain specific ubiquitin-binding domains^18,39–42^. Accordingly, this phenomenon can stabilize transient protein–protein interactions that are crucial for activating downstream signaling. Next, we produced a catalytically inactive TRIM45 mutant, C29A (TRIM45-C29A), that lacks E3 ligase activity^18^. Co-IP assays demonstrated that TRIM45-C29A lost its ability to bind to TAB2, revealing that the E3 ligase activity of TRIM45 is critical for its association with TAB2 (Fig. 5c). Moreover, TRIM45-C29A failed to increase TAB2 ubiquitination compared to TRIM45-WT overexpression (Fig. 5d). In addition, TRIM45-C29A decreased the TAB2–TAK1 interaction (Fig. 5e). HEK293T cells were cotransfected with plasmids encoding TRIM45-C29A or TRIM45-WT plus the NF-κB luciferase reporter plasmid. A luciferase reporter experiment suggested that TRIM45 overexpression increased NF-κB reporter activity, but TRIM45-C29A reversed this effect (Supplementary Fig. 6). These data suggested that TRIM45 exerts its function by relying on E3 ligase activity. To identify the ubiquitination sites, we generated three TAB2 mutants containing arginine at lysine 619, 644, or 656 (K619R, K644R, or K656R). Co-IP assays suggested that only TAB2-K656R failed to interact with polyubiquitin chains (Fig. 5f). More importantly, compared with TAB2-WT expression, TAB2-K656R expression decreased the TAB2–TAK1 interaction (Fig. 5g). Taken together, these results demonstrated that the E3 ligase activity of TRIM45 is required for its ability to mediate the TAB2–TAK1 interaction, which activates the NF-κB signaling pathway.

### TRIM45 knockdown abolishes the neurotoxic effect of proinflammatory microglia in cocultured neurons

**Fig. 6:**
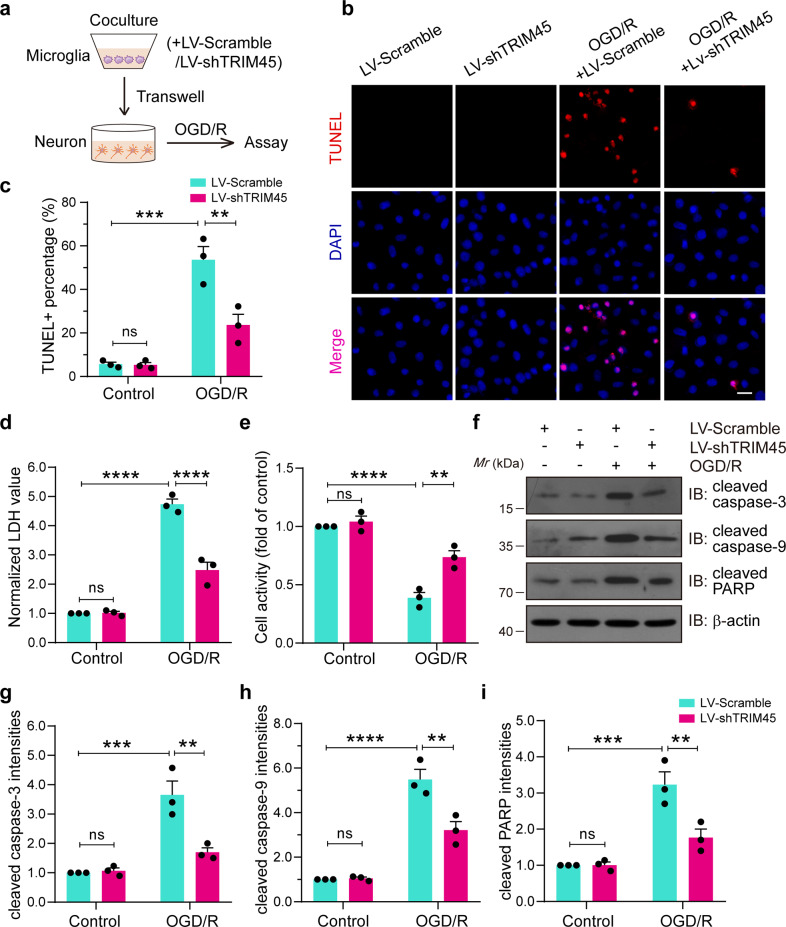
TRIM45 inhibition improves the neurotoxic effect of proinflammatory microglia in cocultured neurons. **a** Primary microglia treated with LV-scramble or LV-shTRIM45 and neurons cocultured via a Transwell system were subjected to OGD/R. Schematic representation as shown above. **b** TUNEL staining indicating the neuronal apoptosis. **c** The percentage of neuronal apoptosis in (**b**). **d** Cell death was quantified by LDH release. **e** Neuronal activity was quantified by CCK-8 assay. **f**–**i** Western blot assays (**f**) and quantification of (**g**–**i**) showing cleaved caspase-3, cleaved caspase-9, and cleaved PARP levels in neurons. Data are presented as the mean ± S.E.M. from at least three independent experiments and were analyzed by two-way ANOVA followed by Tukey’s post hoc test. ns, no significance. **P < 0.01, ***P < 0.001, ****P < 0.0001.

We conclude that TRIM45 increases proinflammatory cytokine and chemokine expression in microglia after OGD/R, which is accompanied by increased cerebral inflammation (Figs. 1, 2). To further confirm whether TRIM45 knockdown could directly protect against neuronal death induced by ischemic stroke through microglial regulation, we transfected microglial cells with LV-TRIM45. Accordingly, we generated a microglia-neuron coculture system (Fig. 6a). TUNEL staining indicated that TRIM45 knockdown in microglia reduced neuronal death after OGD/R compared to that in the coculture group with microglia pretreated with scramble (Fig. 6b, c). Moreover, LDH assays revealed that TRIM45 knockdown in microglia reduced LDH release (Fig. 6d). We next performed CCK-8 assays to determine neuronal viability. The data showed that TRIM45 knockdown increased neuronal viability after OGD/R (Fig. 6e). Immunoblotting assays demonstrated that TRIM45 shRNA in microglia obviously reduced the expression of proapoptotic molecules (Fig. 6f), such as cleaved caspase-3, cleaved caspase-9, and cleaved PARP (Fig. 6g–i), in neurons. These data suggested that TRIM45 inhibition in microglia decreases neurotoxic effects against ischemic neuronal death.

### TRIM45 knockdown in microglia preserves neurobehavioral function following cerebral I/R injury

**Fig. 7:**
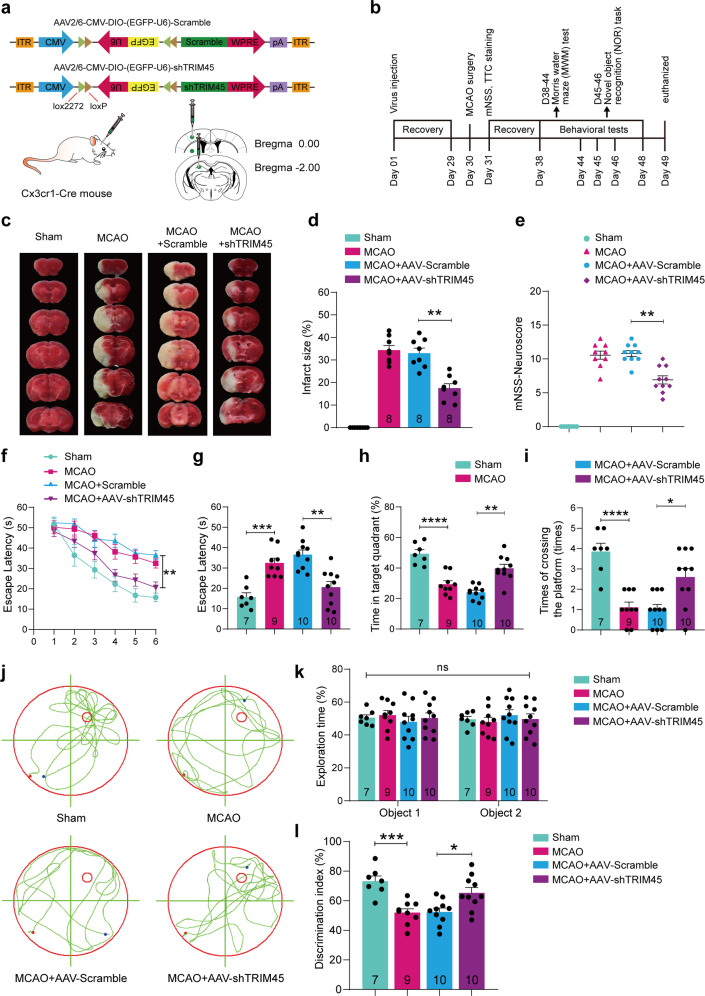
TRIM45 inhibition is neuroprotective during stroke. **a** Experimental design for microglia-specific knockdown of wild-type TRIM45. AAV2/6 vectors encoding CMV-DIO-EGFP-U6-shTRIM45 (or CMV-DIO-EGFP-U6-Scramble) were stereotactically injected into the hippocampal CA1 region, cerebral cortex and striatum of Cx3cr1-Cre mice. **b** Schematic diagram presenting the experimental procedure. **c**, **d** Mouse brains were stained with 2,3,5-triphenyl-2H-tetrazolium chloride (TTC) 24 h after ischemic stroke (**c**), and the infarct volume was quantified (**d**). **e** Neurological deficit scores after ischemic stroke were analyzed using the modified neurological severity score (mNSS). **f** The mean escape latency of the Morris water maze test during Days 1–6 of testing. **g** The latency to reach a hidden platform on Day 6. **h** The time (in seconds, s) spent in the target quadrant during the probe trials on Day 7. **i** Number of times crossing the target platform location during the probe trials on Day 7. **j** Representative swimming traces on Day 7. **k**, **l** Novel object recognition (NOR) test, the exploration time of the mice in the familiarization phase (**k**), and the percentage of time exploring the novel object in the test phase (**l**). *n* = 7–10 mice per group. Statistical differences in (**f**) were determined by RM ANOVA followed by Tukey’s post-hoc test. Data in (**e**, **i**) were assessed by the Kruskal–Wallis nonparametric test, followed by Dunnett’s post hoc test. Data in (**k**) were analyzed by two-way ANOVA followed by Tukey’s post hoc test, and all others were analyzed by one-way ANOVA followed by Dunnett’s post hoc test. Numbers in bars, numbers of mice. Data are presented as the mean ± S.E.M. n.s. for *P* > 0.05, *P < 0.05, **P < 0.01, ***P < 0.001 and ****P < 0.0001.

Our in vitro studies suggested that TRIM45 knockdown decreased the microglia-mediated neurotoxic effect of proinflammatory cytokines and chemokines under OGD/R conditions. Therefore, we speculated that TRIM45 knockdown protects against cerebral ischemia in vivo. To this end, we first used a previously reported method to downregulate TRIM45 expression in microglia of the brain^25,43,44^. Therefore, we constructed an adeno-associated virus type 2/6 carrying TRIM45 shRNA or scramble only in cells expressing Cre recombinase driven by the microglia-specific Cx3cr1 promoter. AAV vectors were stereotactically injected into the cerebral cortex, hippocampal CA1 region, and striatum of adult Cx3cr1-Cre male mice, in which Cre recombinase was specifically expressed in microglia (Fig. 7a). To evaluate the efficacy of AAV-shTRIM45, after four weeks of virus injection, we isolated microglia from AAV-injected mouse brains by Percoll density centrifugation. qRT-PCR and Western blot assays showed statistically significant decreases in TRIM45 mRNA and protein levels (Supplementary Fig. 7a–c). Next, mice were subjected to MCAO followed by reperfusion, and we performed histological and behavioral studies at different time points (Fig. 7b).

First, the infarct volume was determined by TTC staining. Interestingly, TRIM45 ablation decreased the infarct volume (Fig. 7c, d). Furthermore, TRIM45 knockdown alleviated the neurological deficits (Fig. 7e). MWM testing was performed to explore spatial learning and memory. The results revealed that the animals pretreated with shTRIM45 spent less time reaching the hidden platform (Fig. 7f, g). Consistently, the TRIM45 knockdown mice spent more time in the targeting quadrant (Fig. 7h) and had more platform crossovers (Fig. 7i) on Day 7 during the spatial probe trials. Representative swimming traces from the probe trials are presented in Fig. 7j. Next, we investigated mouse memory using the novel object recognition (NOR) task. The mice subjected to MCAO spent less time exploring novel objects, whereas the mice with shTRIM45 exhibited a significant preference for the novel object, indicating an improvement in declarative recognition memory (Fig. 7k, l). Thus, we found that decreasing the induction of TRIM45 in microglia might protect against cerebral I/R injury.

## Discussion

The acute inflammatory response and its resolution are imperative for the physiological repair process after damage, while excessive inflammation is related to cerebral ischemia^45^. Inflammatory resolution in the CNS is a complicated, organized, and active process that requires complicated crosstalk between microglia and injured neurons^46^. Excessive microglial activation leads to proinflammatory cytokine and chemokine release, which subsequently results in neuronal apoptosis^47,48^. However, little is known about what brain factors govern the resolution of neuroinflammation and the underlying mechanisms^49^. In the present study, for the first time, we identified TRIM45 as a critical positive modulator of neuroinflammation after cerebral I/R injury. Specifically, TRIM45 expression was clearly upregulated in microglia after ischemic stroke. Accordingly, suppressing TRIM45 markedly decreased proinflammatory cytokine and chemokine production and attenuated OGD/R-induced neuronal apoptosis. Furthermore, microglia-selective knockdown of TRIM45 in vivo exhibited neuroprotective effects, as proven by the reduced infarct area, decreased neurological deficit scores, and improved cognitive function (Fig. 7c–l). Herein, Trim45 might represent a promising therapeutic target for the treatment of ischemic stroke.

NF-κB signaling has been firmly confirmed to accelerate the development of cerebral I/R injury by promoting the release of inflammatory cytokines. Consistent with the previous studies^50^, we found that NF-κB signaling was activated and that proinflammatory mediators were substantially increased in response to ischemic stroke (Fig. 2c–f and Supplementary Fig. 3a, b). Recent studies have reported that TRIM family proteins are critical regulators of innate immune and inflammatory responses. Shibata et al. reported that TRIM45 negatively regulates TNF-α-induced NF-κB-mediated transcription and suppresses NIH3T3 cancer cell proliferation^19^. However, in the present study, we found that TRIM45 expression was strongly enhanced in the ischemic penumbra in a stroke model. Intriguingly, one of the most relevant findings of this study is that TRIM45 functions as a positive regulator of NF-κB signaling in microglia (Fig. 2c–f). We revealed that knockdown of TRIM45 restricted NF-κB proinflammatory signaling activation after cerebral I/R injury.

As the major immune cells in the central nervous system, microglia play a pivotal role in regulating neuroinflammation, which is implicated in the development of cerebral I/R injury. Overactivation of microglia results in excessive release of proinflammatory cytokines and chemokines. In this study, we found that knockdown of TRIM45 prevented the excessive activation of microglia and inhibited the production of proinflammatory cytokines and chemokines, such as IL-1β, IL-6, TNF-α, Cxcl1, and Ccl2 (Fig. 2a, b). There is also evidence showing that microglial phagocytosis plays a canonical role in neurological recovery after brain injury^51,52^. Ischemic stroke causes substantial cell death in the brain, and microglial phagocytosis is involved in the clearance of dead cells and debris. It is unclear whether TRIM45 also plays a role in microglial phagocytosis after cerebral ischemia. Our study only investigated the effect of TRIM45 on proinflammatory cytokine expression and release, and the role of TRIM45 in microglial phagocytosis after cerebral I/R injury will be investigated in the future.

However, as TRIM45 is not only expressed in microglia (Supplementary Fig. 1), we cannot eliminate the possibility that the effect of TRIM45 on ischemic stroke originates from the cooperative effects of multiple cells in ischemic tissue, including several cell types, such as neurons and astrocytes. However, we found that microglia-specific TRIM45-knockdown mice exhibited an ≈88% decrease in TRIM45 expression in cerebral tissue (Supplementary Fig. 7b, c), showing that TRIM45 expression in brain tissue was mainly upregulated in microglia. This finding may also explain why TRIM45 knockdown in microglia results in impressive histological and behavioral outcomes during cerebral I/R injury. However, Cx3cr1-Cre mice express Cre recombinase under the Cx3cr1 promoter in the mononuclear phagocyte system, including the monocyte and macrophage compartments as well as microglia. Therefore, AAV particles may also infect macrophages and infiltrating monocytes, and the effect of AAV particle injections on infiltrating macrophages and monocytes remains to be determined in future studies.

**Fig. 8:**
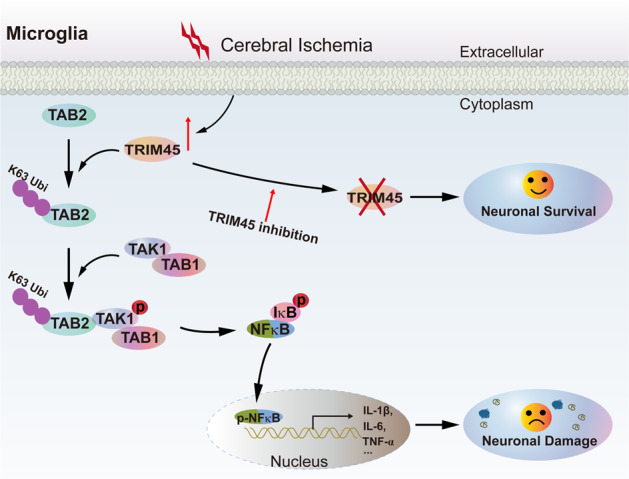
Schematic illustrating the mechanism by which TRIM45 regulates cerebral I/R injury by activating NF-κB signaling in microglia. In response to I/R insults, TRIM45 expression levels in microglia are upregulated. TRIM45 increases the K63-linked ubiquitination of TAB2, followed by recruitment of TAK1/TAB1 to TAB2, thus potentiating the phosphorylation and activation of TAK1. TAK1 sequentially activates the NF-κB signaling pathway, leading to the production of proinflammatory cytokines and ultimately enhancing neuroinflammatory injury. The combined effects of these ischemic signaling cascades lead to profound cerebral injury. Thus, TRIM45 ablation might alleviate neuronal injury via the inhibition of excessive microglial activation after ischemic stroke.

Previous studies have shown that the formation of the TAK1–TAB1–TAB2 complex is important for TAK1 activation^34,35^. However, the mechanism by which TAK1/TAB1 is recruited to TAB2 is unclear. As an E3 ubiquitin ligase, TRIM45 mainly functions as a regulator of ubiquitination of target protein^18s^. In the current study, we showed that TRIM45 conjugates the Lys-63-linked, but not the Lys-48-linked, polyubiquitin chain to TAB2 through its E3 ligase activity, which recruits TAK1/TAB1 and promotes TAK1 autophosphorylation, resulting in NF-κB signaling activation (Fig. 8). These findings were consistent with current reports; Lys-48‐linked polyubiquitin chains promote modified protein degradation, but Lys-63‐linked polyubiquitin chains normally do not lead to degradation of the substrate. Rather, it mediates low-affinity binding of other proteins that contain specific ubiquitin-binding domains^53,54^.

Recent research has reported that microglia-specific knockdown of TAK1 protects against ischemic stroke^55^. This result further proves that TAK1 activation exerts undesirable biological effects during ischemic stroke. Moreover, we found that TRIM45-mediated recruitment and phosphorylation of TAK1 was largely abolished in the absence of TAB2. Furthermore, with a combination of truncated TRIM45 and TAB2 constructs, co-IP assays showed that TRIM45 could bind directly to TAB2 with its RING domain, which was reported to be a critical domain involved in mediating protein–protein interactions^56^. Moreover, the NZF domain of TAB2 through which TRIM45 interacted was also essential for autophosphorylation and activation of TAK1. Therefore, we concluded that TAB2 was involved in TRIM45-dependent TAK1 activation. Nevertheless, our data unambiguously showed that mutant TRIM45 lacking the RING domain critical for the interaction of TAB2 or mutant TAB2 lacking the NZF domain critical for the interaction of TRIM45 failed to activate the TAK1 and NF-κB signaling pathways. Thus, the TRIM45–TAB2 interaction is essential for neuronal loss and adverse stroke outcomes. These findings indicate that targeted regulation of the TRIM45–TAB2 interaction may represent a new strategy to inhibit TAK1 and relieve cerebral I/R injury.

Neuronal death plays a leading role in the progression of cerebral ischemia^57,58^. In this study, we demonstrated that microglia-specific knockdown of TRIM45 had an antiapoptotic effect in neurons. Caspase-3 is a critical factor that results in the development of apoptosis, ultimately leading to DNA fragmentation^59,60^. In addition, TRIM45 was suggested to interact with p53 to enhance apoptosis in glioblastoma cells, preventing tumor growth^18^. Therefore, TRIM45 may have a prominent role in induction of apoptosis. Here, we observed that cleaved caspase-3, caspase-9 and PARP expression was markedly enhanced in neurons subjected to OGD/R, which was consistent with the intensity of apoptosis. However, TRIM45 knockdown in microglia significantly reversed these effects, alleviating neuronal apoptosis. Moreover, downregulated TRIM45 expression clearly improved neuronal activity. These data further demonstrated that TRIM45 ablation inhibited the proinflammatory response induced by excessive microglial activation, thereby blocking neuronal apoptosis and ultimately protecting against ischemic stroke.

In the present study, the mouse MCAO model was used as an acute stroke animal model. We demonstrated that microglia-selective knockdown of TRIM45 reduces the brain ischemic infarct size and diminishes neurological deficit scores. Behavioral tests also showed that knockdown of TRIM45 significantly improved the learning and memory functions of mice subjected to cerebral I/R injury. Because AAV-mediated gene expression requires a certain amount of time in vivo, we pretreated mice with AAV injection four weeks before MCAO operation in this study; thus, more detailed work will be needed to verify the therapeutic effect post-treatment. However, alternative methods to quickly decrease endogenous TRIM45 activity or block its interaction with TAB2 may be a therapeutic strategy for poststroke treatment. Further study is still required in the future.

In summary, our results indicated for the first time that TRIM45 knockdown in microglia is a novel strategy for protecting the cerebrum after I/R injury by inhibiting the microglia-mediated neuroinflammatory response and neuronal apoptosis. This effect is dependent on TRIM45–TAB2 interactions, TAB2 ubiquitination, the formation of the TAK1–TAB1–TAB2 complex and TAK1 autophosphorylation. Therefore, targeting TRIM45 and/or its interaction with TAB2 may provide promising strategies for reversing cerebral I/R injury and possibly other types of neuroinflammatory diseases and disorders.

## Supplementary information

Supplementary data
