# Supplementary material for: TRIM45 causes neuronal damage by aggravating microglia-mediated neuroinflammation upon cerebral ischemia and reperfusion injury
Source: Exp Mol Med. 2022 Feb 25;54(2):180–93. doi: 10.1038/s12276-022-00734-y (PMC8894463; doi:10.1038/s12276-022-00734-y)
Supplement: Supplementary file 1 — Supplementary data [file 12276_2022_734_MOESM1_ESM.pdf]

Supplementary Information

TRIM45 causes neuronal damage by aggravating microglia-mediated neuroinflammation upon cerebral ischemia and reperfusion injury

Qian Xia[1], Gaofeng Zhan[1], Meng Mao[2], Yin Zhao[3], Xing Li[1*]

[1]Department of Anesthesiology, Tongji Hospital, Tongji Medical College, Huazhong University of Science and Technology, Wuhan 430030, Hubei Province, China

[2]Department of Neurobiology, School of Basic Medicine, Tongji Medical College, Huazhong University of Science and Technology, Wuhan 430030, Hubei Province, China

[3]Department of Ophthalmology, Tongji Hospital, Tongji Medical College, Huazhong University of Science and Technology, Wuhan 430030, Hubei Province, China

*Corresponding author:

Xing Li, Department of Anesthesiology, Tongji Hospital, Tongji Medical College, Huazhong University of Science and Technology, Wuhan 430030, Hubei Province, China.

E-mail: lixing88@hust.edu.cn

Supplementary Materials and Methods

Co-culture of neuron and microglia

A co-culture transwell system was used to segregate neuronal cells and microglia cells. Cells were cultured in two loculi segregated by a 0.4 μm semi-permeable membrane to allow cytokines to diffuse between the two chambers. First, the neurons were cultured in the lower compartments of the transwell, while the microglia cells were cultured in the upper chamber using the same procedure described above. After Lentivirus transfected into microglia cells for 48 hours infection, the supernatants were replaced with free fresh medium and the transwell insert was moved to the neurons. The microglia and neurons were co-cultured for 24 h. Then, cells were subjected to OGD/R treatment.

Immunofluorescence

Brain sections and microglia cell slides were washed with PBS 3×5 min, and fixed with 4% paraformaldehyde (PFA) for 15 min at room temperature, and then incubated with 0.1% Triton X-100 for 10 min. The slides were blocked by 5% bovine serum albumin (BSA) (Sigma-Aldrich, Shanghai, China) in PBS for 1 h at room temperature. Slides were incubated with anti-TRIM45 (1:50; Abcam, Cambridge, UK) at 4 °C overnight; then washed slides with PBS for three times. The probed slides were subsequently incubated with FITC-conjugated secondary antibody for 2 h at room temperature. Finally, we stained the slides with 4′, 6-diamidino-2- phenylindole (DAPI; Sigma-Aldrich) at room temperature for 5 min. All images were then examined with a fluorescence microscope (IX73, Olympus, Tokyo, Japan).

RNA interference experiments

Human TRIM45 shRNA plasmids were obtained from GenePharma (Shanghai, China). The target sequence for TRIM45 (GenBank NM_025188.4) shRNA no. 1 was 5′-GCCTTGTTTGCATACAGTTTG-3′, no. 2 was 5′-GCTCAGGAAGCTGAACAAAGT-3′, no. 3 was 5′-GGTGGAGTGAAGGCTTTAACC-3′.

Plasmid construction

The human full-length TRIM45, MyD88, TRAF2, TRAF6, TAB1, TAB2, TAK1, IKKβ, p65 coding sequence was cloned into pFlag-CMV2, the TRIM45 coding sequence was cloned into pHA-CMV2, TAB2 coding sequence was cloned into pMyc-CMV2, and TAB1 coding sequence was cloned into pHis-CMV2 using recombinase connection method. Subsequent mutants were produced by recombination with Trelief SoSoo Cloning Kit Ver.2 according to the manufacturer′s instructions (TSINGKE, Beijing, China). Briefly, subsequent mutants (TRIM45-ΔRING, amino acids 1-57 deleted; TRIM45-ΔB-box, amino acids 131-226 deleted; TRIM45-ΔCC, amino acids 240-355 deleted; TRIM45-ΔFLMN, amino acids 391-580 deleted; TAB2-ΔCUE, amino acids 18-50 deleted; TAB2-(50-532), amino acids 1-50 and 533-693 deleted; TAB2-ΔCC, amino acids 533-619 deleted;

TAB2-ΔNZF, amino acids 666-693 deleted; TAB2-Δ619-666, amino acids 619-666 deleted) were generated. For site-directed mutagenesis, all TAB2 mutants were constructed by a Quickchange® Site-Directed Mutagenesis Kit (Stratagene) using synthetic oligonucleotides containing mutations in the corresponding positions to generate lysine-to-arginine mutation. TRIM45-C29A mutant was constructed in the corresponding positions to generate Cysteine-to-Alanine mutation. All constructs were confirmed by DNA sequencing analysis (performed by Sangon Biotechnology, Shanghai, China).

Luciferase reporter assays

HEK293T cells were seeded in 96-well plates and transfected at 80% confluence with plasmids expressing Scramble or TRIM45-shRNA, and a NF-κB luciferase reporter plasmid (NF-κB-Luc) (firefly luciferase plasmid, Promega, Madison, WI, USA) together with a phRL-TK plasmid (renilla luciferase plasmid, Promega) by using the Fugene transfection reagent (Roche Diagnostics, Basel, Switzerland). Cells were stimulated with OGD/R treatment or exogenous overexpression of MyD88, TRAF2, TRAF6, TAB2, TAB1/TAK1, IKKβ or p65. 24 h after transfection, cells were collected and assayed for luciferase activity with the Dual-Luciferase Reporter Assay System in accordance with the product manual (Promega). Luciferase activity was then examined by normalizing according to the renilla luciferase activity induced by phRL-TK transfection. All transfections were performed in triplicate.

Neurological Score

A modified neurological severity score (mNSS) was used to determine the neurological dysfunction after 24 hours of surgery. The mNSS contains beam balance tests (score 0 to 6), motor tests (including flexion of forelimb, flexion of hindlimb and head movement, score 0 to 6), and reflexes absent & abnormal movements (score 0 to 2). Accumulated points of 1 to 4 indicate mild, 5 to 9 points indicates moderate, and 10 to 14 points indicates severe injury. The neurological function was evaluated by an independent researcher who is blind to the experiment.

Supplementary Figures and Legends

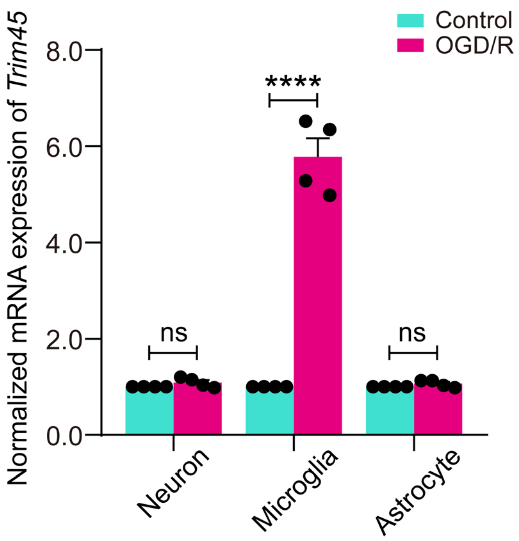

Supplementary Fig. 1 The expression of TRIM45 in primary cultured microglia, astrocytes, and neurons. qRT-PCR was conducted to examine the mRNA level of TRIM45 in primary cultured microglia, astrocytes, and neurons. Values were displayed as mean ± SEM. ****$p < 0.0001$.

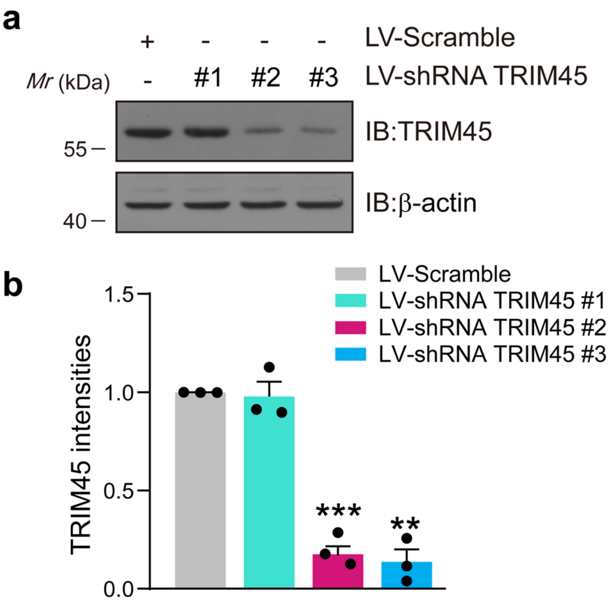

Supplementary Fig. 2 The interference efficiency of LV-shTRIM45 against mouse TRIM45. (a) Effect of LV-shTRIM45 on *Trim45* mRNA expression. qRT-PCR was conducted to examine the mRNA level of TRIM45 in microglial cells pretreated with lentivirus encoding shTRIM45 vector and the quantitative analysis of mRNA levels of TRIM45. (b) Western blotting was conducted to examine the expression level of TRIM45 in microglial cells infected with LV-shTRIM45. Data are representative of three independent experiments. Values were displayed as mean ± SEM, ***$p < 0.001$.

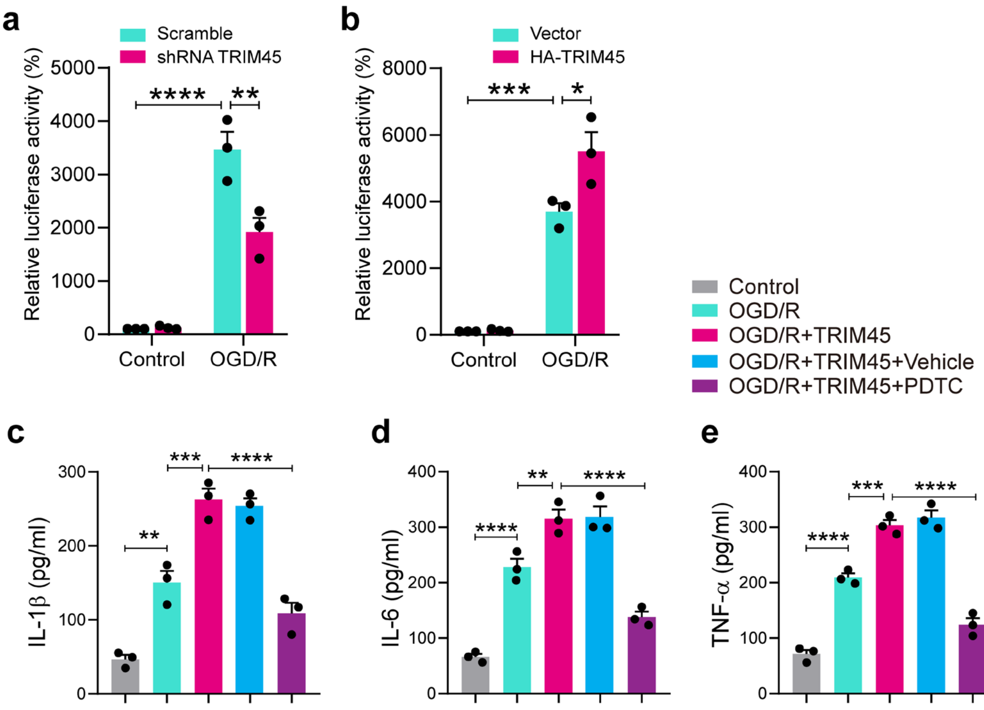

Supplementary Fig. 3 TRIM45 promoted NF-κB signaling pathway activation. (a, b) Luciferase activity in HEK293T cells transfected with plasmids encoding the luciferase reporter for NF-κB together with shTRIM45 (a) or HA-tagged wild-type TRIM45 (b). Data are presented relative to Renilla luciferase activity. (c-e) Primary microglial cells were pretreated with or without 100 μM PDTC for 1 h after being injected with LV-TRIM45 for 48 h, and then subjected to OGD/R. The concentrations of IL-1β (c), IL-6 (d) and TNF-α (e) in the culture media were measured by ELISA. Data are representative of three independent experiments. Values were displayed as mean ± SEM. *p <0.05, **p < 0.01, ***p < 0.001 and ****p < 0.0001.

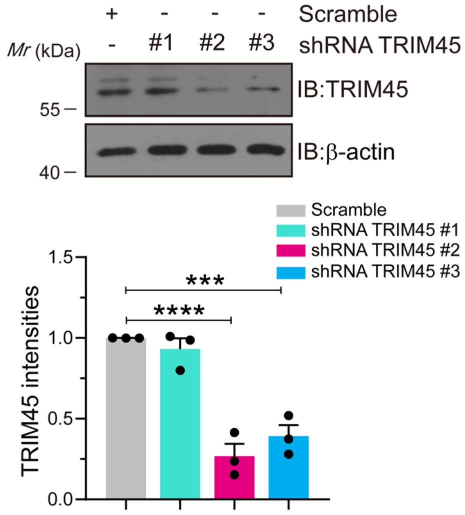

Supplementary Fig. 4 The interference efficiency of shTRIM45 against human TRIM45. HEK293T cells were transfected with plasmids encoding TRIM45 shRNAs. Western blotting was conducted to examine the interference efficiency of shTRIM45. Data are representative of three independent experiments. Values were displayed as mean ± SEM. ***p < 0.001 and ****p < 0.0001.

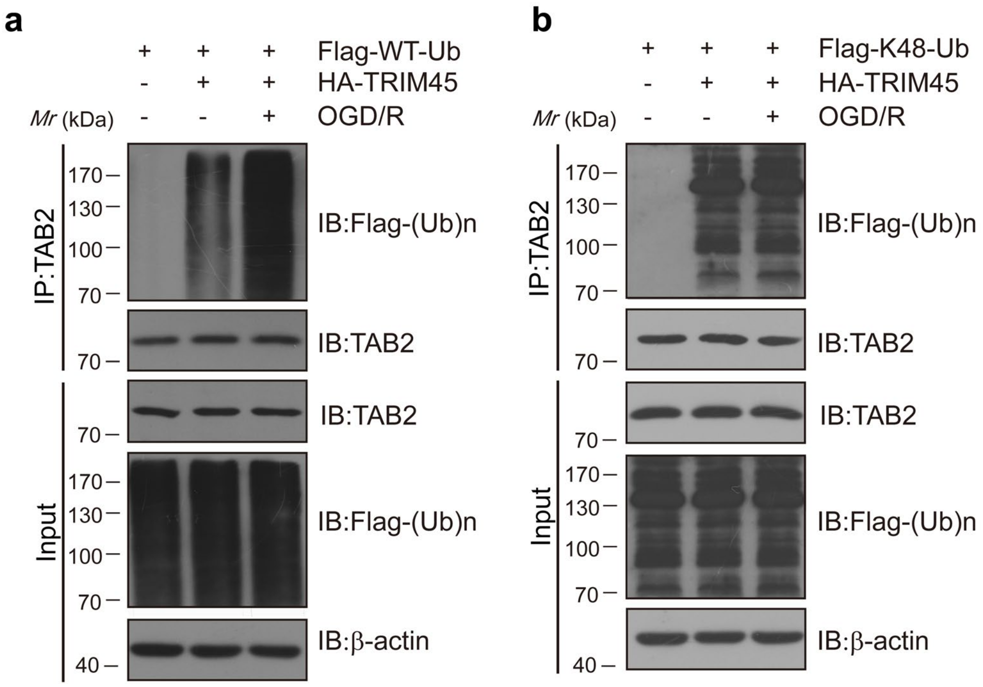

Supplementary Fig. 5 TRIM45 induced polyubiquitination of TAB2 but not with K48 linkage. (a, b) HEK293T cells were transfected with plasmids encoding HA-tagged TRIM45 and Flag-tagged wild-type ubiquitin (Flag-WT-Ub) (a), or Flag-tagged K48 only ubiquitin (Flag-K48-Ub) (b) with or without OGD/R treatment. The cell extracts were collected for IP with anti-TAB2 beads, followed by IB analysis with the indicated antibodies.

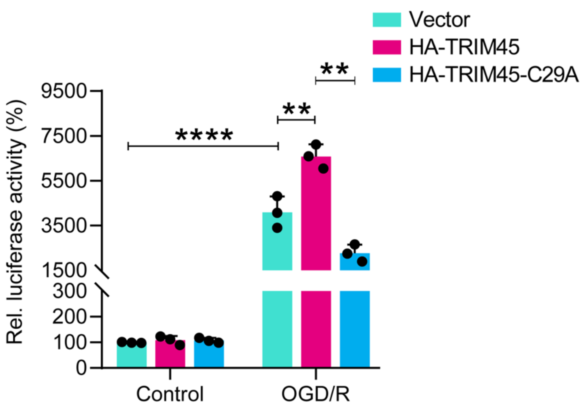

Supplementary Fig. 6 TRIM45 promoted NF-κB signaling pathway activation depending on its E3 ligase activity. Luciferase activity in HEK293T cells transfected with plasmids encoding the luciferase reporter for NF-κB together with HA-tagged wild-type TRIM45 or TRIM45-C29A. Data are presented relative to Renilla luciferase activity. Data are representative of three independent experiments. Values were displayed as mean ± SEM. ** $p < 0.01$, and **** $p < 0.0001$.

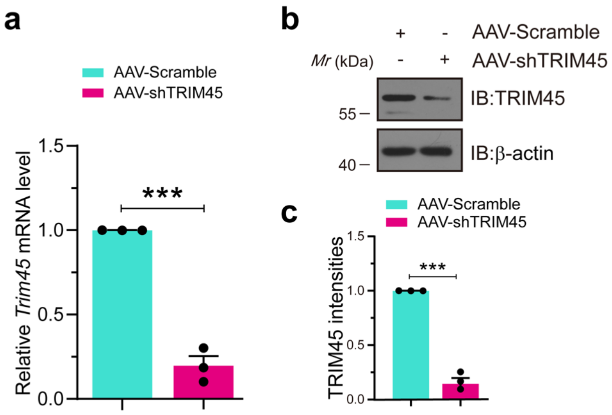

Supplementary Fig. 7 The interference efficiency of AAV2/6-shTRIM45. CMV-DIO-EGFP-U6-shTRIM45 (or CMV-DIO-EGFP-U6-Scramble) were stereotactically injected into hippocampus CA1 region, cerebral cortex and striatum of Cx3cr1-Cre mice. After four weeks, the total proteins of hippocampus CA1 region, cerebral cortex and striatum were harvested. (a) qRT-PCR was conducted to examine *Trim45* mRNA level. (b) Western blotting was conducted to examine the protein level of TRIM45. (c) The data were statistically analyzed in (B). Data are representative of three independent experiments. Values were displayed as mean ± SEM. $^{***}p < 0.001$.

Supplementary Table 1. Antibodies employed in this study

Antibody	Species	Type	IB	IF	Source	Identifier
HA	Mouse	Mono-	1:1000		Santa Cruz	sc-7392
Flag	Mouse	Mono-	1:2000		Santa Cruz	sc-166355
Myc	Mouse	Mono-	1:1000		Santa Cruz	sc-40
TRIM45	Mouse	Poly-	1:1000	1:200	Abcam	ab169036
β-actin	Mouse	Mono-	1:1000		Santa Cruz	sc-47778
NF-κB p65	Rabbit	Mono-	1:1000	1:200	Cell Signaling	#8242
α-tubulin	Mouse	Mono-	1:2000		Santa Cruz	sc-8035
Histone H3	Rabbit	Mono-	1:2000		Cell Signaling	#4499
Iba1	Rabbit	Poly-		1:500	Wako	#019-19741
TAK1	Rabbit	Poly-	1:1000		Cell Signaling	#4505
Phospho-TAK1	Rabbit	Poly-	1:1000		Cell Signaling	#9339
TAB2	Rabbit	Poly-	1:1000		Abcam	ab222214
Ubiquitin	Rabbit	Poly-	1:1000		Abcam	ab7780
cleaved caspase-3	Rabbit	Mono-	1:1000		Cell Signaling	#9664
cleaved caspase-9	Rabbit	Mono-	1:1000		Cell Signaling	#20750
cleaved PARP	Rabbit	Mono-	1:1000		Cell Signaling	#5625

Abbreviations: IB, Immunoblotting; IF, Immunofluorescence.

Supplementary Table 2. Primers used in this study

Primer name	Primer sequences (5'- 3')	
	Forward	Reverse
**Quantitative RT-PCR primers**		
Trim45	AAGATGTCAGAAATCAGGA	GCATCAGAGC GCCACGGTCC
Il-1β	GAAAGACGGCACACCCAC	TGTGACCCTGAGCGACCT
Il-6	TCTCTGGGAAATCGTGGAA	GATGGTCTTGGTCCTTAGCC
Tnf-α	ACGGCATGGATCTCAAAGAC	AGATAGCAAATCGGCTGACG
Cxcl1	GAGCTTGAAGGTGTTGCCCT	CGCGACCATTCTTGAGTGTG
Ccl2	GCAGGTCCCTGTCATGCTTC	GTGGGGCGTTAACTGCATCT
β-actin	CCTTCTTGGGTATGGAATCCTG	CAATGCCTGGGTACATGGTG
**Genotyping primers**		
Cx3cr1 Cre	CAACGAGTGATGAGGTTCGCAAG	ACACCAGAGACGGAAATCCATCG
